# Nicotine Changes the microRNA Profile to Regulate the FOXO Memory Program of CD8^+^ T Cells in Rheumatoid Arthritis

**DOI:** 10.3389/fimmu.2020.01474

**Published:** 2020-07-14

**Authors:** Caroline Wasén, Caroline Ospelt, Alessandro Camponeschi, Malin C. Erlandsson, Karin M. E. Andersson, Sofia Töyrä Silfverswärd, Steffen Gay, Maria I. Bokarewa

**Affiliations:** ^1^Department of Rheumatology and Inflammation Research, Institute of Medicine, Sahlgrenska Academy, University of Gothenburg, Gothenburg, Sweden; ^2^Department of Rheumatology, Center of Experimental Rheumatology, University Hospital Zurich, Zurich, Switzerland; ^3^University of Zurich, Zurich, Switzerland; ^4^Sahlgrenska University Hospital, Gothenburg, Sweden

**Keywords:** rheumatoid arthritis, microRNA, FOXO signaling pathway, programmed cell death 1 (PD-1), memory T cell, CD8^+^ T cell

## Abstract

**Objective:** Smoking suppresses PD-1 expression in patients with rheumatoid arthritis (RA). In this study, we assess if smoking changed the epigenetic control over CD8^+^ T cell memory formation through a microRNA (miR) dependent mechanism.

**Methods:** Phenotypes of CD8^+^ T cells from smokers and non-smokers, RA and healthy, were analyzed by flow cytometry. A microarray analysis was used to screen for differences in miR expression. Sorted CD8^+^ cells were *in vitro* stimulated with nicotine and analyzed for transcription of miRs and genes related to memory programming by qPCR.

**Results:** CD27^+^CD107a^−^CD8^+^ T cells, defining a naïve-memory population, had low expression of PD-1. Additionally, the CD27^+^ population was more frequent in smokers (*p* = 0.0089). Smokers were recognized by differential expression of eight miRs. Let-7c-5p, let-7d-5p and let-7e-5p, miR-92a-3p, miR-150-5p, and miR-181-5p were up regulated, while miR-3196 and miR-4723-5p were down regulated. These miRs were predicted to target proteins within the FOXO-signaling pathway involved in CD8^+^ memory programming. Furthermore, miR-92a-3p was differentially expressed in CD8^+^ cells with naïve-memory predominance. Nicotine exposure of CD8^+^ cells induced the expression of miR-150-5p and miR-181a-5p in the naïve-memory cells *in vitro*. Additionally, nicotine exposure inverted the ratio between mRNAs of proteins in the FOXO pathway and their targeting miRs.

**Conclusions:** Smokers have a high prevalence of CD8^+^ T cells with a naïve-memory phenotype. These cells express a miR profile that interacts with the memory programming conducted through the FOXO pathway.

## Introduction

MicroRNA (miR) are short RNA strands of ~22 nucleotides with gene regulating functions. The human genome includes 2,654 mature miRs annotated in the miR database miRbase v22 (1). They influence the expression of specific genes by guiding the RNA-induced silencing complex (RISC) to complementary strands of messenger RNA (mRNA), often located in the 3′ untranslated region. Depending on the degree of complementary binding between the miR and the targeted mRNA, the complex may degrade the targeted mRNA, or simply block its translation. Thus, the same miR sequence could target many mRNAs, and one mRNA may be under control of several miRs. Transcriptional suppression of a target gene by an individual miR, however, has been suggested to be relatively mild. The suppressive effect of mouse miR-223 on target genes rarely exceeded 33% according to data from a knock-out experiment (2). The interaction between miRs and mRNAs can be experimentally determined, and these validated interactions are listed in the DIANA-TarBase database (3). Computer algorithms, for example the DIANA-microT-CDS algorithm, can be used to predict the binding of a strand of miR to mRNA (4).

MiRs that regulate immune cells have a strong connection to autoimmune disorders. A meta-analysis from 2019 reported over 400 studies investigating the miR expression in the peripheral blood from patients with the canonical autoimmune disease rheumatoid arthritis (RA) (5). A summary of 31 studies that were of sufficient quality, highlighted only a limited number of differentially expressed miRs in connection to RA. Specifically, miR-16, miR-24, miR-26, miR-155, and miR-223 were upregulated, and miR-21 was downregulated. These studies included measurements of miRs directly in plasma, in total peripheral blood mononuclear cells (PBMC), and CD4^+^ T cells and B cells isolated from the peripheral blood and in synovial fibroblasts. However, the miR signature in CD8^+^ T cells have not been investigated, despite the emerging role of this cell population in the RA pathogenesis (6).

Furthermore, the relation of T cell miR expression and smoking, the major environmental and preventable risk factor for development of RA, is currently unknown. A large prospective cohort including of 5,023 participants from the Framingham Heart Study, investigated the effects of smoking on whole-blood miRs (7). Five miRs were negatively associated with a smoking history and one miR, mir-345-5p, had a positive association. Another study analyzed the effect of smoking on a selection of 11 miRs in human plasma of healthy donors (8). The study concluded that miR-21 was significantly downregulated in plasma of smokers. A different study reported that miR-29b and RNU6-2 was upregulated in plasma of smokers and miR-223 was downregulated (9). Yet another study compared the miR expression in sera of 10 smokers and 10 non-smokers and detected differential expression of as many as 105 miRs, of which 73 were down regulated (10). The impact of smoke reduction on miR expression in monocytes of the peripheral blood has also been investigated in teenagers (11). Twenty-five miRs with differential expression were identified 3 months after smoke cessation; 19 of those were upregulated.

We have previously reported that nicotine from cigarette smoke is a critical regulator of cytotoxic CD8^+^ T cells. In the cytotoxic CD8^+^ T cell population, smoking limited the expression of the co-inhibitory receptor programmed death-1 (PD-1) (12) and the soluble form of its ligand (PD-L1) (13), which functions as brakes on the immune system to facilitate the resolution of inflammatory responses and protect against autoimmunity. These findings contribute to our understanding of the connection between smoking and RA, and support a role for CD8^+^ T cells in RA. However, the underlying mechanisms of this phenotypic change of CD8^+^ T cells in response to smoking and nicotine have not been fully elucidated. In the present work we ask if smoking, or direct stimulation of nicotinic receptors, may influence the phenotype of CD8^+^ T cells by changing the miR expression profile.

## Materials and Methods

### Patients

MiR microarray, phenotype, and transcriptional profile of the peripheral blood CD8^+^ T cells were analyzed in 17 RA patients [average age 58.8 (45–76) years], disease duration 14.4 (2–44) years, and in 10 healthy controls [average age 57.9 (49–80) years]. Nicotine stimulation was performed in CD8^+^ T cells from 25 RA patients, disease duration 8.6 years (1–30 years). The study was approved by the Swedish Ethical Review Authority (WSAS, diary no. 593-08; RA, diary no. 659-11). The study was carried out in accordance with the Declaration of Helsinki and patients gave informed written consent prior to participation.

### CD8^+^ T Cell Isolation

Blood samples were collected from 15 RA patients and eight healthy controls for microarray analysis, and 25 RA patients for the *in vitro* stimulation with nicotine. Peripheral blood mononuclear cells (PBMC) were isolated through gradient centrifugation using Lymphoprep (Fresenius Kabi, Oslo, Norway). CD8^+^ T cells were enriched in the PBMC using Stemcell negative selection human CD8^+^ T cell enrichment kit according to the manufacturer's instructions. The purity of CD8^+^ cells were 81–92%, as determined by flow cytometry.

### Cell Culture and *in vitro* Stimulations

Isolated CD8^+^ T cells were cultured at a density of 400,000–1000,000 cells/ml in 24-well cell culture plates in in RPMI medium including 10% fetal bovine serum (Sigma-Aldrich, St. Louis, MO, USA), 4 mM Glutamax (Gibco), 50 mM β-mercaptoethanol (Gibco), and 50 mg/mL gentamycin (Sanofi-Aventis, Paris, France), a temperature of 37°C and an atmosphere of 5% CO_2_. Cells were stimulated either by coating the culture plates with anti-CD3 antibodies (72 h), which provides stimulation by binding to the T cell receptor, or by adding phorbol 12-myristate 13-acetate/ionomycin (PMA/IM, 30 and 500 nM, 2 h), which provides unspecific cell stimulation by enhancing calcium influx and protein kinase C activation. 10 μM nicotine (Sigma) was used for 48 h nicotine exposure experiments.

### Enzyme-Linked Immunosorbent Assay

IFN-γ levels in cell culture supernatants were assessed with PeliKine compact human IFN-gamma (Sanquin, Amsterdam, Netherlands). Supernatants were diluted 1:20 and analyzed with a lower detection limit of 20 pg/ml.

### Flow Cytometry

Blocking was performed with human normal gamma-globulin (Beriglobin, CSL Bhering, King of Prussia, Pennsylvania, USA). Cells were first incubated with antibodies binding to extracellular proteins. Cytofix/cytoperm (BD Biosciences) was used to fix the cells for 20 min, followed by an overnight incubation with a blocking/permeabilization solution. The following day the cells were stained with antibodies binding to intracellular proteins. The following anit-human antibodies were used: Per-Cp-anti-CD8 cloneSK1 (BD), PE-anti-CD27 clone L128 (BD), FITC-anti-CD27 clone M-T271 (BD), PE-anti-CD107a clone eBioH4A3 (eBioscience), BV421-anti-PD1 clone EH12.1 (BD), APC-anti-CD62L (BD), and PE-Cy7-anti-CD45RA (BD). The samples were analyzed with a BD FACSCanto II instrument and the FACSDiva software (BD Biosciences), data analysis was performed in the FlowJo software (Tree Star, Ashland, OR, USA). Isotype controls and fluorescence minus one (FMO) were used to facilitate gating.

### RNA Isolation and Quantitative Polymerase Chain-Reaction (qPCR)

RNA was prepared from cell lysates with the microRNA Purification Kit (Norgen Biotec Corp, Canada) according to manufacturer's protocol. The concentration and quality of the RNA were evaluated with a NanoDrop spectrophotometer (Thermo Scientific, USA) and Experion™ RNA StdSens Analysis chip (Bio-Rad Laboratories, Hercules, CA, USA). Complementary deoxyribonucleic acid (cDNA) synthesis was performed using High Capacity cDNA Reverse Transcription Kit (Applied Biosystems, Foster city, CA) or miScript II RT Kit (Qiagen, Valencia, CA, USA). Gene transcription was assessed using SYBR Green qPCR Mastermix, miScript SYBR® Green PCR Kit (SABiosciences, Qiagen) for mRNA or TaqMan assays for miR, and plates were read with a ViiA™ 7 Real-Time PCR (Applied Biosystems). Primers for CYBR® Green assays were purchased from Sigma and the sequences are presented in Supplementary Table 1. Melting curves for each reaction were performed between 60 and 95°C. The results were calculated as a fold change compared to controls with the ΔΔCT-method in relation to glyceraldehyde- 3-phosphate dehydrogenase (GAPDH) for mRNA and RNU6b for miR.

### Microarray

Total RNA was extracted from CD8^+^ T cells of 23 females, 15 were RA patients and eight were healthy controls (HC). To obtain the amount of RNA sufficient for the microarray analysis, some of the samples were pooled from 1 to 3 individuals according to RA diagnosis and smoking status. Clinical information for each sample combination is presented in Table 1. The samples were divided into tubes so that each tube contained ~15 μl. They were then concentrated down to 5 μl in the Biovac 060, iLMVAC after which, they were pooled together again and further concentrated down to 5 μl. Concentrations where then measured on the Dropsense 96 (Trinean). One sample were diluted to a final amount of RNA was 250 ng before being run on the microarray platform. miR expression was analyzed on a 3D microarray platform Toray by TATAA Biocenter (Gothenburg). The amount of RNA added to the microarray varied between 23 and 250 ng. The maximum input volume of 2.2 μl of each sample was then mixed with miRNA spike (Cat No TRT-XR304, Toray) and further labeled, hybridized and washed according to the instruction manual from Toray (H-M-R miRNA protocol 4-Plex V3 using Toray miRNA Labeling kit (Cat No TRT-XE211) and Human miRNA Oligo chip 4plex (2), based on miRBase 21 (Cat No TRT- XR520, Toray). The intensity of each miRNA was analyzed with the 3D-Gene Scanner 3000 (Toray) with auto gain, auto focus, and auto analysis settings, all per manufacturer's instructions. Quality control was performed on all 15 samples based on the QC report from the instrument. Every “spot” where the intensity exceeded the average value of the noise plus two standard deviations were considered a true signal and were subjected to background subtraction. Two samples (ID 9 and 10) were excluded from further analysis due to the very low number of miRs exceeding the background threshold.

**Table 1:** Characteristics of CD8^+^ T cell donors analyzed by microRNA microarray.

**Sample ID**	**Diagnosis**	**Smoker**	**Disease duration**	**MTX, mg/w**	**cDMARD**	**Biologics**	**Prednisolone, mg/d**
1	RA	No	5	10	MTX	TNFi	NA
	RA	No	8	25	MTX	TNFi	NA
	RA	No	9	0	SLZ	TNFi	NA
2	RA	Yes	23	15	MTX	CTLA4	NA
	RA	Yes	5	10	MTX	TNFi	NA
3	HC	Yes	NA	0	No	No	NA
	HC	Yes	NA	0	No	No	NA
4	HC	No	NA	0	No	No	NA
	HC	No	NA	0	No	No	NA
5	RA	Yes	31	25	MTX	CTLA4	NA
6	HC	No	NA	0	No	No	NA
7	RA	No	13	20	MTX	aCD20	NA
8	HC	Yes	NA	0	No	No	NA
*9*	*RA*	*Yes*	*10*	*25*	*MTX*	*TNFi*	*NA*
*10*	*HC*	*No*	*NA*	*0*	*No*	*No*	*NA*
11	RA	Yes	11	25	SLZ+PQ	No	5
	RA	Yes	44	0	No	TNFi	5
12	HC	Yes	NA	0	No	No	NA
13	RA	No	2	15	MTX	TNFi	NA
	RA	No	22	12.5	MTX	TNFi	NA
14	RA	Yes	7	0	SLZ	TNFi	NA
15	RA	Yes		20	MTX	TNFi	NA
16	RA	No	11	20	MTX	No	NA

*The CD8^+^ T cells from 23 donors were pooled into 16 samples based on diagnosis and smoke status. RA, patients with rheumatoid arthritis; HC, healthy controls; MTX, methotrexate; SLZ, sulfasalazine; PQ, Plaquenil; TNFi, TNFa inhibitors (infliximab, golimumab, etanercept); CTLA4, CTLA4-immunoglobulin fusion protein (abatacept); aCD20, anti-CD20 antibodies (rituximab); RNA, ribonucleic acid; NA, not applicable. Sample 9 and 10 are shaded in gray because they were excluded from the analysis*.

### Pathway Analysis

We used the online software DIANA-miRPath v.3 Using DIANA-miRPath v.3 (14) to find pathways targeted by differentially expressed miRs. This software uses the algorithm DIANA-microT-CDS (4) for an *in-silico* prediction of the interactions between the differentially expressed miRs and complementary sequences of mRNA. Each miR can be complementary to several mRNA. Next, DIANA-miRPath uses the Kyoto Encyclopedia of Genes and Genomes (KEGG) database to find pathways in which the predicted genes are enriched.

### Statistics

Comparison between groups was conducted by the Mann-Whitney *U*-test using GraphPad Prism v.7 for Mac OS X, or Microsoft Excel using the Real Statistics Resource Pack software v 5.4, Copyright (2013–2018) Charles Zaiontz (www.real-statistics.com). Data was presented as boxplots, representing the median and the inter-quartile range, whiskers indicate range.

The top 125 miRs with the highest average expression from the microarray analysis were log2 transformed and normalized through cyclic loess normalization using *R*, R studio and the LIMMA (Linear models for microarray data) package. LIMMA was further used to analyze differential expression. Heatmaps and clustering were performed using the gplots and hclust packages.

## Results

### CD8^+^ T Cells of Smokers Gain a Naïve-Memory Phenotype

**Figure 1:**
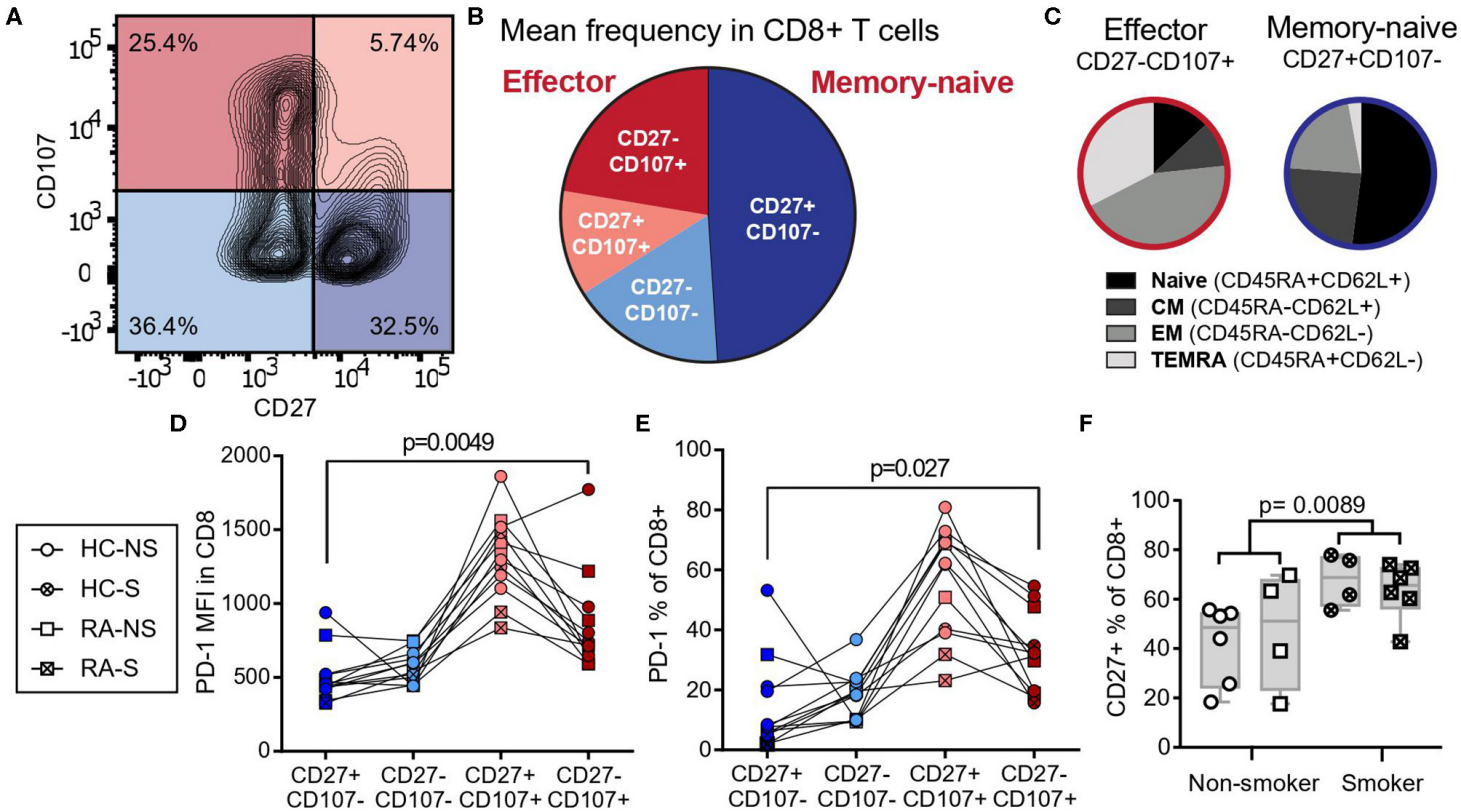
CD8^+^ T cells of smokers gain a naïve-memory phenotype. CD8^+^ peripheral blood monocytes from RA patients (*n* = 5, two smokers) and healthy controls (*n* = 7, two smokers) were isolated and cultured for 72 h with aCD3 stimulation. **(A)** Gating strategy for peripheral blood CD8^+^ cells by expression of CD27 and CD107a. **(B)** The mean frequency of CD8^+^ populations based on their expression of CD27 and CD107a, CD27^+^CD107a^−^ defined naïve-memory cells and CD27^−^CD107a^+^ defined effector cells (CD27^−^CD107^−^: 17.05% ± 2.40 (mean ± standard error of the mean); CD27^+^CD107^−^: 48.98% ± 4.70; CD27^−^CD107^+^: 22.35% ± 2.98; CD27^+^CD107^+^: 11.61% ± 1.76). **(C)** Separate experiment demonstrating the distribution of CD27 and CD107a in T cell subpopulations based on CD45RA and CD62L expression. **(D,E)** The median fluorescence intensity (MFI, **D**) and frequency **(E)** of PD-1 expressing cells within CD8^+^ populations. **(F)** The frequency of CD27^+^ cells in smokers (*n* = 10 of which six have RA) and non-smokers (*n* = 10 of which four have RA). The *p*-values were calculated with Mann-Whitney *U*-test or Wilcoxon matched- pairs signed rank test. The boxes represent median and quartiles, the whiskers represent the range. CM, central memory cells; EM, effector memory cells; TEMRA, terminally differentiated effector memory cells re-expressing CD45RA; NS, non-smoker; S, smoker; HC, healthy control; RA, patients with rheumatoid arthritis.

We have previously reported that smoking limits the expression of the co-inhibitory receptor PD-1, which marks the exhausted state of CD8^+^ T cells (12). To further map the phenotypic changes induced by smoking, we divided CD8^+^ T cells isolated from the peripheral blood of female RA patients and healthy controls into four populations based on the expression of CD107a, a degranulation marker expressed by cytotoxic cells releasing effector molecules, and CD27, a co-stimulatory receptor constituently expressed in naïve and memory cells (Figure 1A). The largest population was positive for CD27 and negative for CD107a, while the population positive for CD107a and negative for CD27 was smaller (Figure 1B) Classic markers to distinguish the human T cell subsets naïve, central memory, effector memory, and terminally differentiated effector memory cells re-expressing CD45RA (TEMRA) are CD45RA and CD62L. To investigate how the populations defined by CD27 and CD107a related to these markers, blood from healthy donors were co-stained for CD27, CD107a, CD45RA, and CD62L. The CD27^+^CD107a^−^ population of human CD8^+^ T cells included primarily naïve (CD45RA^−^CD62L^+^) or central memory (CD45RA^+^CD62L^+^) cells (Figure 1C) and are referred to as naïve-memory. The population of CD27^−^CD107a^+^ cells included primarily effector T cells (CD45RA^−^CD62L^−^) and TEMRA (CD45RA^+^CD62L^−^, Figure 1C) and are referred to as effector cells. Investigating the PD-1 expression in the subsets defined by CD107a and CD27, we found that CD27^−^CD107a^+^ effector cells had higher expression levels (median fluorescent intensity) and frequency of PD-1 (*p* = 0.0049 and *p* = 0.027, Figures 1D,E) compared to CD27^+^CD107a^−^ naïve-memory cells. Furthermore, the CD27^+^ population was significantly larger in smokers (*p* = 0.0089, Figure 1F), potentially explaining the loss of PD-1 expression in CD8^+^ cells of smokers.

### microRNAs Differentially Expressed in Smokers Regulate the PI3K-AKT-FOXO Signaling Pathway

**Figure 2:**
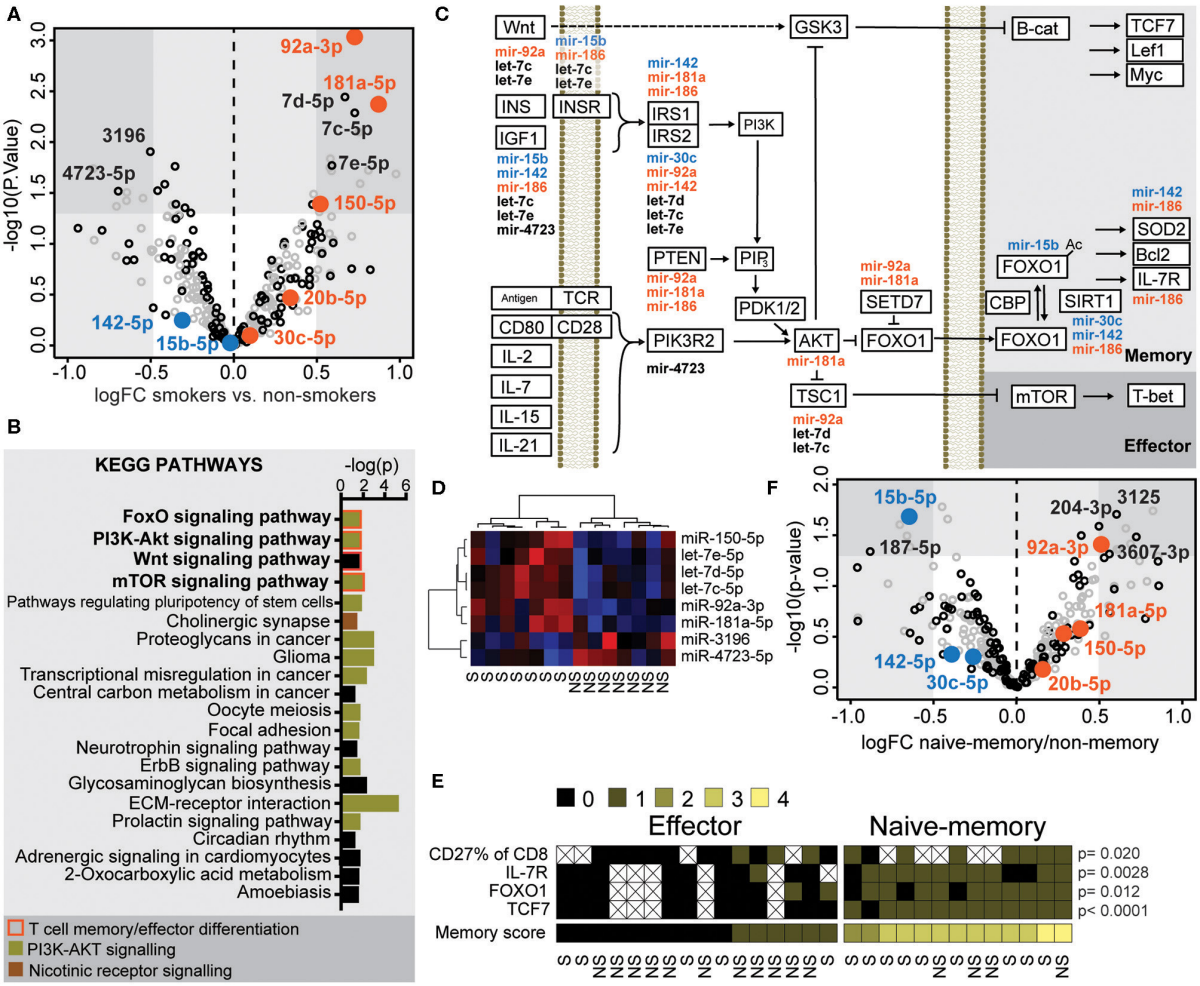
microRNAs differentially expressed in smokers regulate the PI3K-AKT-FOXO signaling pathway. Isolated CD8^+^ T cells from human peripheral blood were activated with 30 nM PMA and 500 nM ionomycin for 2 h. **(A)** Fold change of miR expression in smokers compared to non-smokers. MiRs with complete data are included; those exceeding an average microarray signal intensity of 80 are presented in black. MiRs included in further analyses are highlighted in color orange and blue for up- or downregulated, respectively. **(B)** Kegg pathways that involve the miRs that were significantly down- or upregulated in smokers. Pathways of T cell effector/memory differentiation, PI3K-AKT signaling or nicotine signaling are indicated by color as indicated with color key. **(C)** A graphic map of the Kegg pathways indicated with bold in **(B)**. MiRs predicted to inhibit expression of proteins involved in these pathways are placed adjacent to the protein. **(D)** Heatmap and clustering of miRs differentially expressed in smokers. Clustering was based on Ward's minimum clustering method and distances were calculated with Spearman correlations. **(E)** A scoring system was constructed to differentiate between naïve-memory and effector phenotype. A frequency of CD27^+^CD8^+^ cells above the median of all samples, or a higher than median expression of IL7R, FOXO1, or TCF7 mRNA were rewarded with one point each. Samples with at least two points were considered to have a naïve-memory phenotype. *P*-values between the two groups were calculated with the Mann-Whitney *U*-test. The smoking status of each individual is indicated below. **(F)** Volcano plot of the fold change of miRs in samples with naïve-memory vs. effector phenotype according to the scoring system in **(E)**. S, smoker; NS, non-smoker. An X indicates missing data.

We suggested that a shift in the miR environment might underlie the phenotypic changes seen in CD8^+^ cells exposed to nicotine from cigarette smoke (12). To study this, we analyzed RNA of CD8^+^ T cells isolated from the peripheral blood of 23 women (Table 1) and measured expression of 2,656 miRs using a 3D microarray. In total, 125 miRs had an average signal exceeding 80 arbitrary units and were included in the analysis (Figure 2A, black circles). Analysis of differential expression with the R package LIMMA (15) demonstrated that six miRs were increased in smokers, with a log fold change (logFC) > 0.5 and a *p*-value < 0.05 (Figure 2A). These miRs were miR-181a-5p, let-7c-5p, miR- 92a-3p, let-7d-5p, let-7e-5p, and miR-150-5p. Two miRs were down regulated in smokers, with a logFC < −0.5. These miRs were miR-3196 and miR-4723-5p (Figure 2A). Unsupervised clustering demonstrated that these miRs correctly distinguished smokers and non-smokers into separate clusters (Figure 2D). Comparing RA patients to healthy controls, the expression of 4 miRs were significantly different. In RA patients, miR-6743, miR-494, and miR-7641 were downregulated with a logFC < −0.5 and miR-6780b-5p was upregulated with a logFC larger than 0.5 (Supplementary Figure 1).

Using the software DIANA-miRPath v.3 (14) and the algorithm DIANA-microT-CDS (4), we estimated potential mRNA targets of the eight miRs differentially expressed in smokers and conducted a pathway analysis to identify which intracellular pathways contained these protein targets. Among 21 KEGG pathways, which had a predicted significant interference from these miRs (Figure 2B), 12 of the pathways were involved in PI3K-AKT signaling. T cell co-stimulation, the common-γ chain cytokines and insulin-like growth factor-1 (IGF-1), all activate Akt and had a central position in the FOXO pathway dependent on PI3K-Akt. The Wnt pathway is indirectly influenced by PI3K-Akt signaling through the Akt-induced inhibition of Glycogen synthase kinase 3 (GSK3) and β-catenin. Together these pathways orchestrate the memory and effector T cell formation (Figure 2C). Interestingly, the differentially expressed miRs in smokers were also predicted to interact with the cholinergic synapse. For example, let-7c-5p and let-7e-5p target the expression of the nicotinic receptor gene CHRNA7, which activate the PI3K/AKT signaling pathway via JAK2. The pathway with the strongest connection to the differentially expressed miRs was the extracellular membrane (ECM) receptor interaction pathway. The miRs were predicted to interact with the integrin subunits α3, α5, αV, α6, α7, β3, and β6, that are involved in biding fibronectin, laminin, and other extracellular matrix proteins. Of these, β6, β3, and αVβ6 are known target genes of the FOXO1 transcription factor (16).

To further investigate the role of these miRs in formation of CD8^+^ memory cells, we set to identify samples with a naïve-memory profile with regard to protein and mRNA levels. We employed flow cytometry data for frequency of CD27^+^CD8^+^ population, and mRNA levels for IL-7R, FOXO1 and TCF7 in CD8^+^ T cells, and a higher-than-median level of each marker was rewarded by one point. CD8^+^ cells with at least two points were included in the naïve-memory group (Figure 2E). From this analysis, we observed that smokers tended to have a naïve-memory phenotype. Indeed, nine of 13 patients with the naïve-memory group were smokers and only five of 16 patients with an effector profile were smokers (Figure 2E, OR 4.95, *p* = 0.067). Comparing samples of the naïve-memory and effector groups, we could see that miR-92a-3p was significantly increased in the naïve-memory group, and miR-15b-5p was significantly decreased (Figure 2F). These changes, in relation to previously observed miR levels in memory T cell differentiation, can be compared in Supplementary Table 2.

### *In vitro* Stimulation With Nicotine Induces miR-181a-5p and miR-150-5p in CD8^+^ T Cells With Naïve-Memory Phenotype

**Figure 3:**
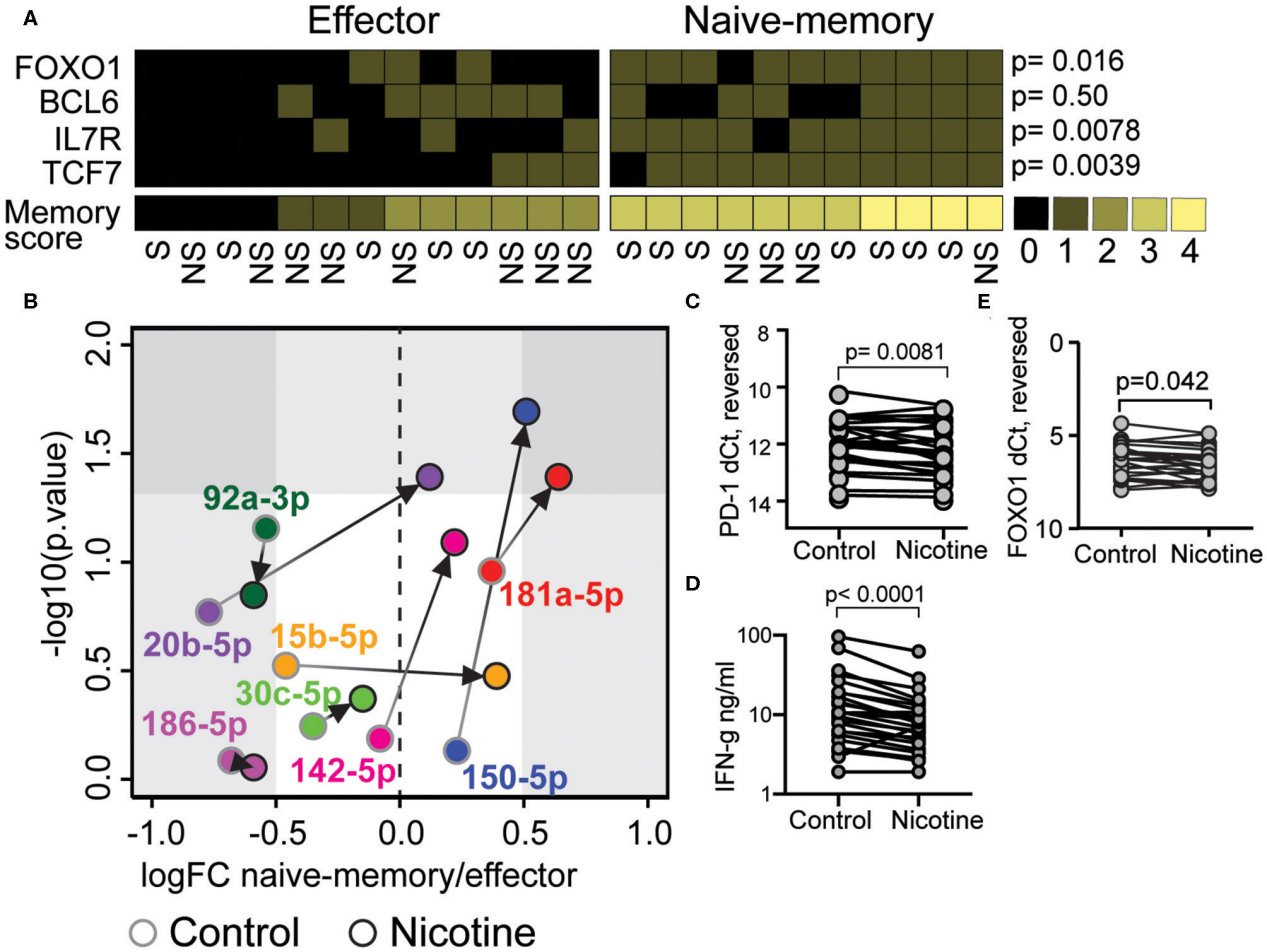
Nicotine stimulation induces micro RNA (miR)-181a-5p and miR-150-5p in CD8^+^ T cell with a naïve-memory phenotype. Isolated CD8^+^ T cells from peripheral blood of rheumatoid arthritis patients were cultured for 48 h in presence of aCD3 stimulation. Messenger RNA (mRNA) and miRs were measured by quantitative PCR. **(A)** A scoring system was constructed to differentiate between naïve-memory and effector phenotype. A higher than median expression of IL7R, FOXO1, BCL6, and TCF7 mRNA were rewarded with one point each. Samples with at least three points were considered to have a naïve-memory phenotype. **(B)** The fold change (FC) of miRs in samples with naïve-memory vs. effector phenotype. Cells were stimulated with aCD3 alone (gray circles) or with an addition of 10 μM nicotine (black circles). **(C,D)** PD-1 mRNA **(C)**, FOXO1 mRNA **(D)**, and IFN-γ secretion **(E)** in cells treated with 10 μM nicotine compared to cells only exposed to αCD3. *P*-values were calculated with Mann-Whitney *U*-test or Wilcoxon matched-pairs signed rank test. S, smoker; NS, non-smoker.

Next, we wanted to investigate if nicotine supported acquisition of the naïve-memory phenotype. CD8^+^ T cells were isolated from peripheral blood of RA patients and stimulated *in vitro* with 10 μM nicotine and aCD3 for 48 h, while the control CD8 cultures were stimulated with aCD3 alone. Using the previously constructed score of mRNA expression for memory markers FOXO1, BCL6, IL7R, and TCF7, we identified control CD8^+^ cultures with the naïve-memory profile (≥3 points). Seven of 11 patients with the naïve-memory profile were smokers and five of 13 patients with effector profile were smokers (Figure 3A, OR 2.8, *p* = 0.41).

We compared the expression of miRs predicted to interact with the FOXO signaling pathway in patients with naïve-memory or effector profiles. In control cultures of CD8^+^ T cells, stimulated with αCD3 alone, there was no significant differences in the measured miRs (Figure 3B). However, when cells received additional nicotine stimulation, the individuals with a naïve-memory profile had significantly higher expression of miR-181a-5p and miR-150-5p (Figure 3B). Interestingly, nicotine stimulation resulted in the reduced levels of PD-1 mRNA (Figure 3C, *p* = 0.0081), reduced levels of FOXO1 mRNA (Figure 3D, *p* = 0.042) and reduced the release of IFN-γ into supernatants (Figure 3E, *p* < 0.0001).

### The Ratio Between FOXO Pathway mRNA and miR Predicted the Response to Nicotine

Since the primary function of miR is to guide degradation of target mRNA, we investigated the relation between miRs and their mRNA targets within the FOXO signaling pathway in CD8^+^ T cells.

**Figure 4:**
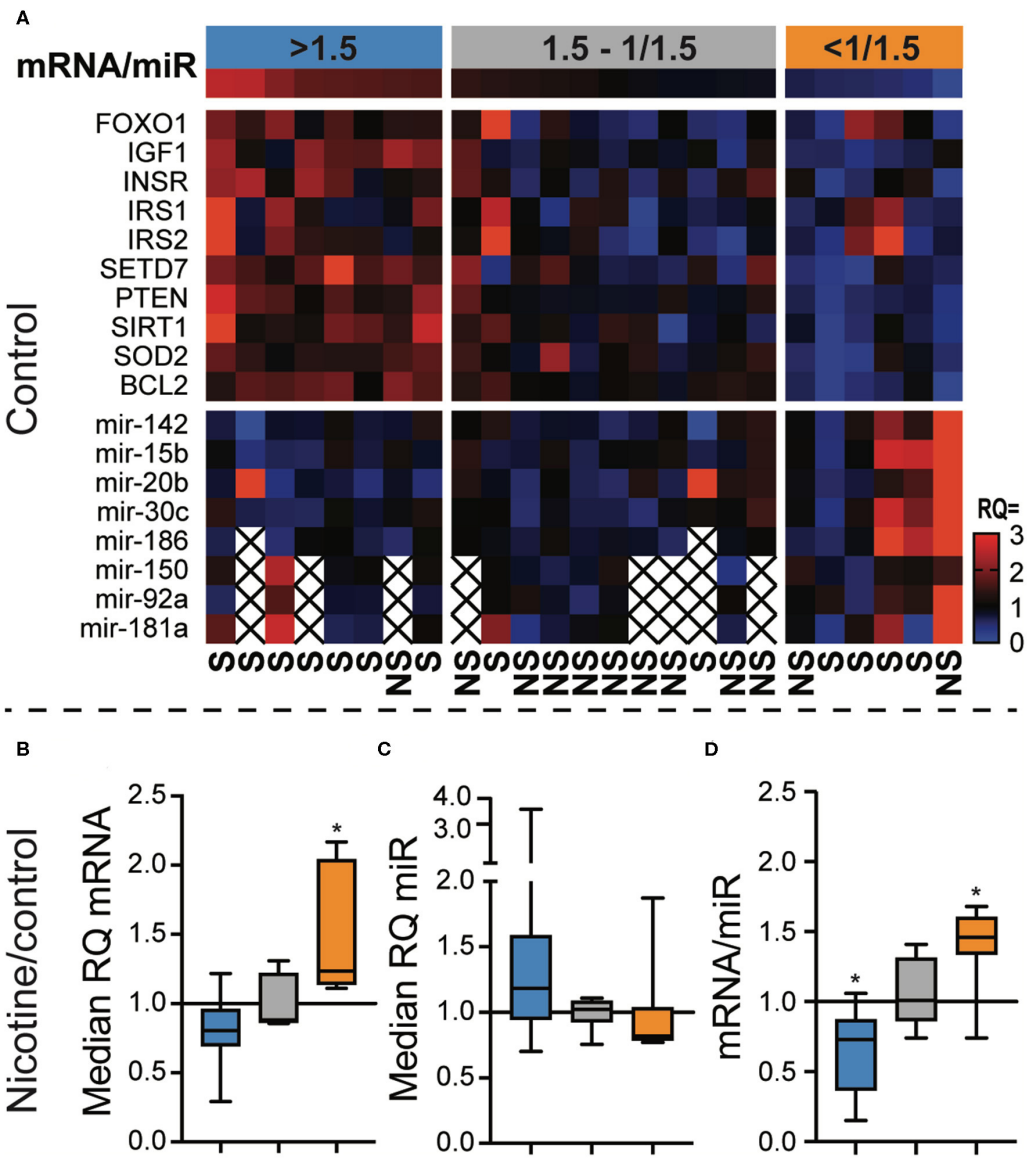
The ratio between FOXO pathway messenger RNA (mRNA) and micro RNA (miR) predicted the response to nicotine. CD8^+^ cells were enriched from peripheral blood monocytes of patients with rheumatoid arthritis. The cells were stimulated with αCD3 antibodies for 48 h and analyzed for their expression of miRs targeting mRNAs of the FOXO signaling pathway. **(A)** Heatmap showing the relative quantity (RQ) of the mRNA and miR in each sample. The samples (columns) were ordered by the mRNA/miR expression ratio. Three groups were defined using a higher cutoff of 1.5 and a lower cutoff of 1/1.5. **(B,C)** The relative quantity of mRNAs **(B)** and miRs **(C)** in cells stimulated with αCD3 and 10 μM nicotine, compared to αCD3 stimulated cells. **(D)** The mRNA/miR ratio after nicotine stimulation in relation to controls. *p < 0.05. S, smoker; NS, non-smoker. An X indicates missing data.

We calculated the relative quantity (RQ) to the median of each individual mRNA and miR in unstimulated, control CD8^+^ cell cultures (Figure 4A). In the next step, we calculated the median RQ of all mRNAs and miRs, and then we calculated the ratio between mRNA and miR. Eight samples had mRNA/miR ratio >1.5, six samples had mRNA/miR ratio <1/1.5, and 11 samples had an intermediate mRNA/miR ratio. CD8^+^ cells with the low or high mRNA/miR ratios were more frequently smokers than the intermediate group (Figure 4A, OR = 16.5, *p* = 0.0048). CD8 cells with the mRNA/miR ratio >1.5 had significantly more frequently naïve-memory profile than patients with lower ratio (OR = 21.0, *p* = 0.0078).

Interestingly, the mRNA/miR ratio in control samples predicted how CD8^+^ cells responded to nicotine exposure. We observed that CD8 with high mRNA/miR ratio in control cultures tended to respond to nicotine by down regulating mRNA (Figure 4B) and upregulating miRs (Figure 4C). Similarly, CD8 cells with a low mRNA/miR ratio responded to nicotine by upregulating mRNA and downregulating miRs. This resulted in a significantly reduced mRNA/miR ratio in CD8 cells that had originally high ratio (*p* = 0.0078) and in an increased mRNA/miR ratio in CD8 cells with an originally low ratio (*p* = 0.031, Figure 4D).

## Discussion

In the present study, we learned that the CD8^+^ T cell population that express low levels of PD-1 belong to the naïve-memory subgroup of T cells and express CD27. The frequency of CD27^+^CD8^+^ T cells was higher in smokers. This was consistent with our previous results demonstrating that mouse CD8^+^ T cells that had lost PD-1 expression due to nicotine exposure would upregulate IL-7R, another marker of the naïve-memory populations (12). In this report, we confirm that nicotine stimulation of CD8^+^ T cells in the experimental setting results in reduced PD-1 mRNA levels. Previous *in vitro* stimulations of mouse splenocytes provided no conclusive evidence to how nicotine regulates the phenotypic changes in CD8^+^ T cells of smokers, although cells exposed to nicotine seemed to have increased levels of the T-bet transcription factor specifically within the PD-1^+^ population (12). Here, we convincingly demonstrate that the population with low levels of PD-1 belongs to the naïve-memory subset expressing also CD27. The frequency of CD27^+^CD8^+^ T cells was higher in smokers. Neither naïve nor memory cells are finally differentiated subsets, and thus, have potential for long-term survival and formation of rapidly proliferating terminally differentiated effector cells upon activation. Effector cells rapidly become unresponsive/exhausted during prolonged antigen exposure. Thus, the naïve-memory cells could presumably have more potential in the chronic autoimmune setting of RA. It was recently postulated that exhausted T cells, despite their high expression of PD-1, were unable to regain their killing capacity after PD-1 inhibition by pharmacological interventions. Instead, a stem cell-like memory population maintained by the transcription factor TCF7 responded to this treatment (17).

Comparing miR expression in smokers and non-smokers, we learned that eight highly expressed miRs were differentially expressed in smokers. These miRs were predicted to target four tightly integrated pathways that all play major role in the memory T cell formation, including the FOXO signaling pathway. We suggested that these miRs could indeed be involved in the formation of CD27^+^ naïve-memory CD8 cells observed in smokers. FOXO1 induces the transcription of memory transcription factor Eomes and inhibits the effector transcription factor T-bet (18). Continuous expression of FOXO1 is required to maintain the survival, renewal, and gene expression profile of the memory subset (19). FOXO1 stimulates the transcription of the memory markers IL-7R and CD62L, that are involved in homing and long-term survival, but negatively regulates effector functions, including the production of interferon-γ and Granzyme B (20, 21). Indeed, we saw that cells stimulated with nicotine reduced their production of IFN-γ, supporting the assumption that smoking induced the naïve-memory phenotype. On the other hand, nicotine stimulation *in vitro* reduced FOXO1 mRNA expression. The strong connection between smoking and RA led us to speculate whether smoking related changes in the immune cell phenotype could contribute to the development of RA. Previous study comparing the transcription levels of FOXO1 in peripheral blood monocytes reported lower levels of FOXO1 mRNA in RA patients compared to healthy controls (22), which was consistent with lower FOXO1 expression in nicotine stimulated cells, but not with an increased abundance of cells with naïve or memory phenotype. Another pathway that is targeted by the differentially expressed miRs is the ECM-receptor pathway, that includes several integrin receptor subunits. Integrins have a direct controlling function over FOXO expression. This provides support to our hypothesis of the valuable part of miRs in development of CD8^+^ memory cells in RA. In addition, αVβ3 provide costimulatory signals to effector CD8^+^ T cells through stimulation by vitronectin, that triggers degranulation and release of cytotoxic effector (23).

Comparing CD8^+^ cells with naïve-memory expression profile with their counterparts with low expression of the same proteins revealed differential expression of miRs associated with the FOXO signaling pathway. Specifically, mir-92a-3p was upregulated in naïve-memory cells and mir-15b-5p was downregulated. These results were rather unexpected. MiR-92a-3p is a member of the miR-17~92 cluster of miRs that plays an important role in memory and effector differentiation. However, over expression of this cluster has been shown to result in impaired memory formation during viral infection (24). In contrast, mir-15b was previously reported to induce the memory state in CD8^+^ T cells and promote their survival (25). Consequent with these findings is the higher expression of mir-150-5p that we see in smokers. Indeed, previous experimental results indicate that miR-150-5p block FOXO mediated memory formation by directly targeting FOXO1. In addition, miR-150-5p knockout mice had increased formation of central memory cells (26). Our results were further strengthened by the fact that *in vitro* stimulation with nicotine induced the relative quantity of mir-150-5b in CD8^+^ cells with a naïve-memory profile. In conclusion, several differentially expressed miRs related to the FOXO pathway have been previously demonstrated to block the memory cell formation rather than to promote it.

On the other hand, mir-181a-5p tell a different story. MiR-181a-5p was significantly more abundant in smokers and in naïve-memory CD8 cells following nicotine stimulation *in vitro*. From the theoretical perspective, mir-181a-5p inhibits the expression of AKT, PTEN, and IRS1, which lead to the increased FOXO1 activity and memory differentiation (illustrated in Figure 2C). These interactions have been experimentally confirmed (27), and increased acetylation of the FOXO1 gene has also been suggested via inhibition of SIRT1 (28). This is further supported by recent experimental reports demonstrating that mir-181a deficiency leads to reduced recall responses after infection (29), a key feature of memory T cells. Interestingly, mir-181a could also lower the activation threshold of T cells by blocking phosphatases downstream the T cell receptor (30), as opposed to PD-1 which is known to increase the activation threshold.

Next, we aimed to gain a better understanding of the relationship between the gene transcription and miR expression. Thus, we analyzed the ratio between mRNA belonging to the FOXO pathway, and a selection of their targeting miRs. Our results convincingly show that the relative amount of mRNA and miR are discordant within the majority of the samples. In addition, this relation predicted the CD8^+^ T cell response to nicotine. Only samples with a mRNA/miR ratio higher or lower than a factor 1.5 responded significantly to nicotine when stimulated. Initially low miR levels resulted in a decrease of mRNA/miR ratio, shifting the mRNA/miR ratio toward miR expression to the opposite of the initial pattern. Interestingly, patients whose cells responded to nicotine stimulation were often smokers. It is possible that previous exposure to nicotine through cigarette smoking would stimulate the expression of nicotinic receptors on these cells, making them more responsive to nicotine.

A limitation to our analysis was the isolation of CD8^+^ T cells by the use of magnetic beads, resulting in a cell purity of ~80–90%. Thus, other cell types may to some degree have contributed to the observed miR profile. To avoid confounding our analysis by miRs from other cell types, we applied a cutoff that removed miRs with a low average expression from the analysis of differential expression between smokers and non-smokers. Furthermore, it is not given that differences in T cell phenotypes observed in smokers and non-smokers are mediated by nicotine. Cigarette smoke contains a broad variety of substances with toxic properties. However, nicotine is one of the few substances in cigarette smoke that is absorbed and systemically distributed to a high degree, and lymphocytes do have receptors for nicotine (31). Expression of α4-and β4-nAChR subunits have been demonstrated in T cells isolated from human peripheral blood, which are subunits of both major types of high affinity nicotinic acetylcholine receptors. In addition, the low affinity receptor subunit α7-nAChR was inducible by stimulation by αCD3 and/or nicotine (31). Stimulation of these receptors with nicotine lead to a dose dependent effect on calcium influx when the cells were treated with nicotine in concentrations between 0 and 50 μM in addition to αCD3. We have previously demonstrated that 10 μM is sufficient to alter the expression levels of transcription factors central for memory vs. effector differentiation in CD8^+^ T cells (12). Since interaction with extracellular matrix and integrins is shown here to be potential mediators of miR dependent processes, we may not exclude the role of external and heterogeneous cell-to-cell interactions for miR expression by CD8^+^ cells. Aiming to substitute a lack of such stimulation, we activated CD8 cells by ligation of CD3. This does not always substitute of specific integrin stimulus.

In conclusion, we demonstrate that smoking promotes a naïve-memory phenotype in the CD8^+^ T cell population resulting in the loss of PD-1 expression. Additionally, we suggest that smoking influences the miR expression pattern of miRs that are predicted to interact with the FOXO signaling pathway, including the increased expression of mir-181a-5p.

## Data Availability Statement

The datasets generated for this study can be found in the GEO database with accession number GSE146393 (https://www.ncbi.nlm.nih.gov/geo/query/acc.cgi?acc=GSE146393).

## Ethics Statement

The studies involving human participants were reviewed and approved by The Swedish ethical review authority. The patients/participants provided their written informed consent to participate in this study.

## Author Contributions

CW: experimental design, data acquisition, data analysis, and manuscript preparation. CO: experimental design, data analysis, and manuscript preparation. AC, ME, and KA: data acquisition and manuscript preparation. SS: study coordination and manuscript preparation. SG and MB: study design, experimental design, and manuscript preparation. All authors contributed to the article and approved the submitted version.

## Conflict of Interest

The authors declare that the research was conducted in the absence of any commercial or financial relationships that could be construed as a potential conflict of interest.
