# Supplementary material for: Nicotine Changes the microRNA Profile to Regulate the FOXO Memory Program of CD8+ T Cells in Rheumatoid Arthritis
Source: Front Immunol. 2020 Jul 14;11:1474. doi: 10.3389/fimmu.2020.01474 (PMC7381249; doi:10.3389/fimmu.2020.01474)
Supplement: Supplementary file 1 [file Table_1.DOCX]

**Supplementary table 1. Primers**

| **Target** | **Forward primer** | **Exon (F)** | **Reverse primer** | **Exon (R)** |
| --- | --- | --- | --- | --- |
| TCF7 | TGACCTCTCTGGCTTCTACTCC | exon 5 | GGTACACCAGAACCTAGCATCAAG | exon 6 |
| SOD2 | GACAAACCTCAGCCCTAACG | exon 3 | GAAACCAAGCCAACCCCAAC | exon 4 |
| SETD7 | TGAGAGGACCGCACTTTATG | exon 4 | CAGCAACATAAACCCTTTCTGA | exon 5, 6 |
| SIRT1 | GCTGTGAAATTACTGCAAGAGTG | exon 3 | GTGACAGAGAGATGGCTGGA | exon 5 |
| INSR | AGATGACAACGAGGAGTGTGG | exon 2 | AGCCGTGTGACTTACAGATGG | exon 3 |
| IGF1 | GCTCTTCAGTTCGTGTGTGG | exon 2 | ATCCACGATGCCTGTCTGAG | exon 3 |
| FOXO1 | ACGAGTGGATGGTCAAGAGC | exon 1 | GCACACGAATGAACTTGCTG | exon 2 |
| IGF1R | GGAGCGAAGACTGAGTTTGAG | exon 1 | TGATAGTCGTTGCGGATGTC | exon 2 |
| IRS1 | GTTTCCAGAAGCAGCCAGAG | exon 1 | GGATTTGCTGAGGTCATTTAGG | exon 2 |
| IRS2 | CTTCTTGTCCCACCACTTGA | exon 1 | TGAAACAGTGCTGAGCGTCT | exon 2 |
| IL7R | GCAATATATGTGTGAAGGTTGGAG | exon 3 | TCCCGATAGACGACACTCAG | exon 4 |
| Bcl6 | CCCTACAAATGCGAAACCTG | exon 3 | CACAGGGATAGGGCTTCTCA | exon 2 |
| PTEN | GCAGAAAGACTTGAAGGCGTA | exon 2 | GCAATTAAATTTGGCGGTGT | exon 4, 5 |
| BCL2 | GGGTCATGTGTGTGGAGAGC | exon 1 | GCCAGGAGAAATCAAACAGAGG | exon 2 |
