# Supplementary material for: Nicotine Changes the microRNA Profile to Regulate the FOXO Memory Program of CD8+ T Cells in Rheumatoid Arthritis
Source: Front Immunol. 2020 Jul 14;11:1474. doi: 10.3389/fimmu.2020.01474 (PMC7381249; doi:10.3389/fimmu.2020.01474)
Supplement: Supplementary file 2 [file Table_2.DOCX]

**Supplementary table 2. Expected vs. observed miR levels in smokers compared to non-smokers, and memory T cells compared to effector T cells.**

|  | **In literature (T cells)** | | **Actual outcome (CD8)** | |  |  |  |
| --- | --- | --- | --- | --- | --- | --- | --- |
| **MiR** | miR levels in memory T cells | T cell miR levels in smokers | miR levels in memory T cells | T cell miR levels in smokers | Ref. |  |  |
| **miR-17~92** | ↓ | Unknown | ↑ | ↑ | Liang (28) | | |
| **miR-150** | ↑ | Unknown | ↔ | ↑ | Liang (28), Ban (24) | | |
| **miR-15b** | ↑ | Unknown | ↓ | ↔ | Liang (28), Zhong (23) | | |
| **miR-142-5b** | ↑ | Unknown | ↔ | ↔ | Liang (28) | | |
| **miR-181a** | ↑ | Unknown | ↔ | ↑ | Kim (27) | |  |
| **miR-20b** | Unknown | Unknown | ↔ | ↔ |  |  |  |
| **miR-30c** | Unknown | Unknown | ↔ | ↔ |  |  |  |
|  |  |  |  |  |  |  |  |
