# Supplementary figures and images for: Nicotine Changes the microRNA Profile to Regulate the FOXO Memory Program of CD8+ T Cells in Rheumatoid Arthritis
Source: Front Immunol. 2020 Jul 14;11:1474. doi: 10.3389/fimmu.2020.01474 (PMC7381249; doi:10.3389/fimmu.2020.01474)

# RA vs. healthy controls

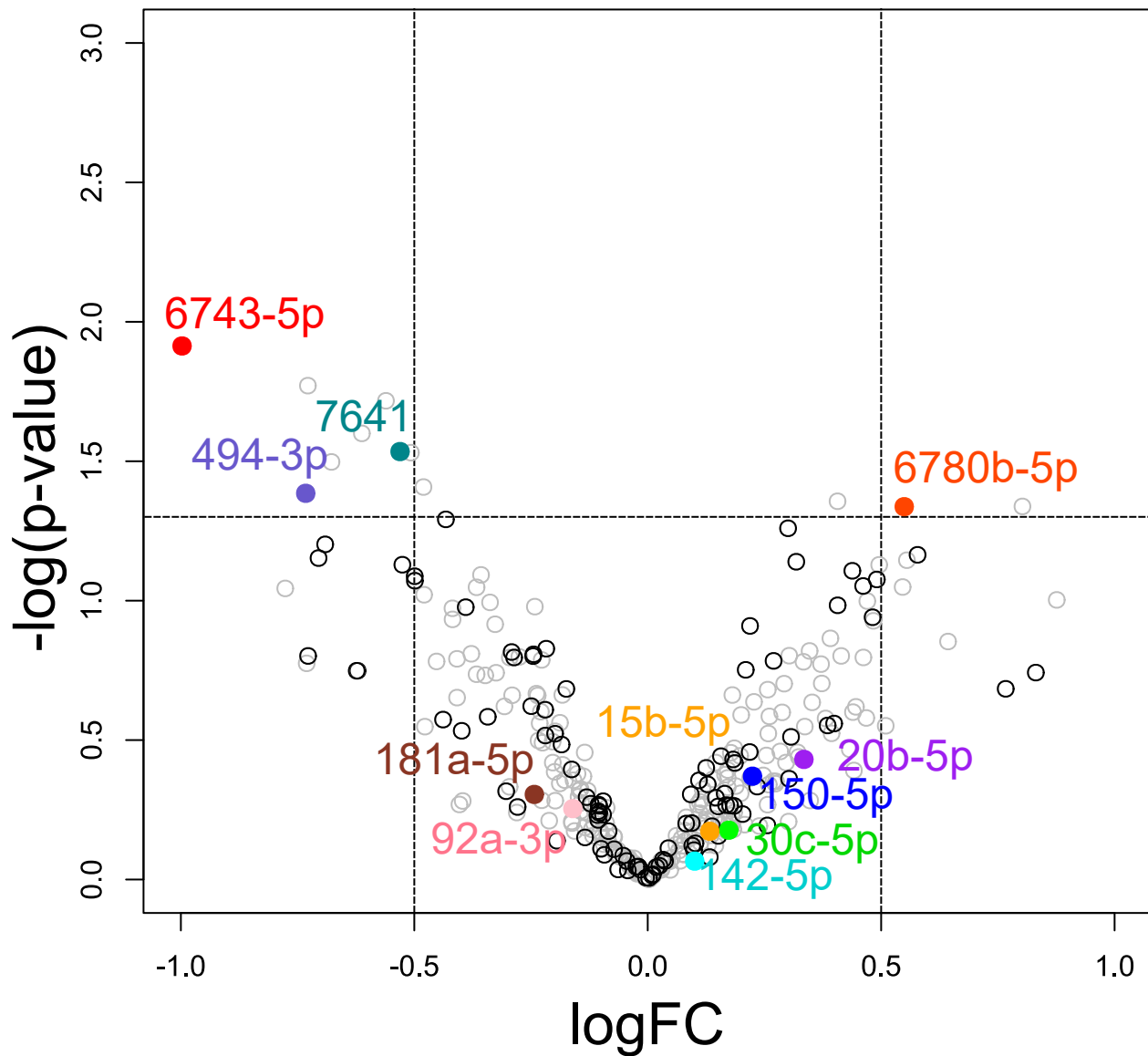

Supplement: Supplementary file 3 [file Data_Sheet_1.PDF]
